# The safety and efficacy of endoscopic approaches for the management of Zenker’s diverticulum: a multicentre retrospective study

**DOI:** 10.1007/s00464-024-11164-4

**Published:** 2024-08-19

**Authors:** Benjamin Norton, Katie Siggens, Apostolis Papaefthymiou, Andrea Telese, Margaret Duku, Alberto Murino, Gavin Johnson, Charles Murray, Borzoueh Mohammadi, Muntzer Mughal, Raf Bisschops, Pradeep Bhandari, Martin Birchall, Rehan Haidry

**Affiliations:** 1 Digestive Diseases & Surgical Institute, Cleveland Clinic London, London, UK; 2 Department of Gastroenterology, University College London Hospital, Euston Road, London, NW1 2BU UK; 3 Department of Gastroenterology, Portsmouth University Hospitals, Portsmouth, UK; 4 Department of Gastroenterology and Hepatology, UZ Leuven. TARGID, Louvain, KU Belgium; 5 Royal National Ear Nose and Throat and Eastman Dental Hospitals, University College London Hospital, London, UK; 6 The Ear Institute University College London, London, UK; 7 Centre for Obesity Research, University College London, London, UK; 8 Division of Surgery and Interventional Science, University College London, London, UK

**Keywords:** Zenker’s diverticulum, Pharyngeal pouch, Peroral endoscopic myotomy, Rigid diverticulotomy, Flexible diverticulotomy

## Abstract

**Introduction:**

Minimally invasive endoscopic options are safe and effective alternatives to surgery for the treatment of symptomatic Zenker’s diverticulum (ZD). However, there is no consensus on the gold-standard approach. We compared the safety and efficacy of Zenker’s peroral endoscopic myotomy (Z-POEM), flexible diverticulotomy (FD), and rigid diverticulotomy (RD) for the management of ZD.

**Methods:**

Patients undergoing treatment for ZD at three UK tertiary referral centres were identified and analysed between 2013 and 2023. Patient demographics, procedural details, clinical success, and 30-day adverse events (AE) were recorded. The primary outcomes were technical and clinical success defined as a fall in Dakkak and Bennett dysphagia score to ≤ 1 without re-intervention.

**Results:**

There was no difference in baseline characteristics amongst 126 patients undergoing intervention (50 RD, 31 FD, 45 Z-POEM). Technical success for RD, FD, and Z-POEM was 80%, 100%, and 100%, respectively (*p* < 0.001). Over a mean follow-up of 11.0 months (95% CI 8.2–13.9), clinical success amongst those treated was 85.3% (RD), 74.1% (FD), and 83.7% (Z-POEM; *p* = 0.48) with recurrence in 17.2% (RD), 20.0% (FD), and 8.3% (Z-POEM; *p* = 0.50). AEs were equivalent between groups (*p* = 0.98). During this time, 11 patients underwent surgical myotomy with low clinical success (36.4%) and high morbidity.

**Conclusion:**

Endoscopic options for the treatment of ZD show equivalent rates of success, but failed RD often led to open myotomy with worse outcomes. Flexible endoscopic modalities are both safe and highly effective treatments that may be considered first-line in experienced centres and should be offered before surgery.

**Graphical abstract:**

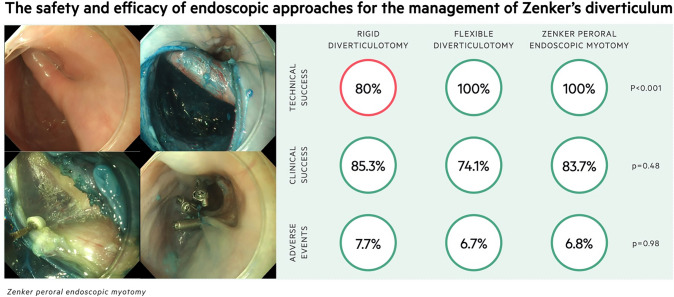

A Zenker diverticulum (ZD), more commonly known as a pharyngeal pouch, is a herniation of the posterior pharyngeal wall that occurs at an area of weakness within the inferior pharyngeal constrictor. The orientation of the muscular fibres in this region creates a triangular gap, known as Killian’s triangle, that is prone to formation of a diverticulum [1]. Clinically, ZD is characterised by oropharyngeal dysphagia and regurgitation of undigested food debris that may occur hours after eating [2]. Patients may experience additional symptoms, including chronic cough, halitosis, globus, hoarseness, and aspiration. ZD is rare before 40 years old and predominantly occurs in elderly patients with a male predominance [3]. Once identified, treatment should be restricted to patients who are symptomatic [1]. The rationale for treatment is that ZD can lead to major complications, and in elderly frail populations, it can predispose to medication ineffectiveness, malnutrition, and life-threatening aspiration pneumonia.

The aim of treatment for ZD is to dissect the septum of the cricopharyngeal muscle by performing a myotomy. This creates a common cavity between the pouch and the oesophageal lumen. Historically, the main treatment has been through open neck surgery, which is associated with high technical success (80–100%), but recurrence is seen in up to 19% and it is associated with significant morbidity and mortality compared to endoscopic techniques [4]. Due to its less invasive nature, rigid endoscopic methods have become prominent, with the use of endoscopic stapling becoming the most used technique over the last two decades. This is associated with a reported clinical success of 88–92% over 27–32 months, recurrence in 10–11%, and complications in up to 7.1% with mortality in 0.3% [5]. One of the drawbacks with this approach has been the less precise nature of the procedure, access to the pouch due to anatomical challenges and deformities of the cervical spine in older patients, rates of complications, and not insignificant conversion to open surgery (11.5%) [6].

Due to the access difficulties and risks of rigid endoscopic approaches, minimally invasive flexible endoscopic options have emerged as successful and safe alternative treatments. Flexible diverticulotomy (FD) is a simple endoscopic procedure that involves mucosal incision followed by septotomy. This is associated with a clinical success rate of 91%, adverse event rate of 11.3%, and recurrence in 10.3% [7]. With the advent of third space endoscopy, an emerging flexible technique is Zenker peroral endoscopic myotomy (Z-POEM). The development of Z-POEM has been from the extrapolation of knowledge gained from over a decade of experience treating achalasia [8]. Z-POEM provides the perceived benefits of a more complete and controlled myotomy, lower risk of perforation and mediastinitis, and lower risk of recurrence, whilst preserving the degree of invasiveness [9, 10].

Despite the evolution in endoscopic treatments for ZD, there is still no consensus on the gold-standard approach with limited data comparing these techniques. Therefore, we conducted a UK-based retrospective cohort study comparing the safety and efficacy of Z-POEM, FD, and rigid diverticulotomy (RD) for the management of ZD.

## Materials and methods

### Study design

We conducted a retrospective, multicentre, cohort study of all consecutive patients undergoing treatment for ZD at three tertiary referral centres (Portsmouth University Hospital, University College London Hospital, and Cleveland Clinic London) in the UK between 2013 and 2023. Patient demographics, co-morbidities, investigations, procedure details, and follow-up data were extracted using electronic health records. Follow-up data on adverse events and clinical success were obtained from health records as part of routine clinical care. Patients who had previously consented to be part of the Cleveland Clinic London (CCL) prospective POEM registry were contacted via telephone.

### Treatment groups

Patients were divided into three groups based on the endoscopic treatment for ZD, which included

- Rigid diverticulotomy (RD)
- Flexible diverticulotomy (FD)
- Zenker peroral endoscopic myotomy (Z-POEM)

All procedures were performed by clinicians highly experienced in the management of ZD. Flexible endoscopic approaches were performed by two independent endoscopists (R.H, P.B) and a single otolaryngology surgeon (M.B) performed all RD procedures. Both endoscopists have at least ten years of experience in the management of pharyngeal pouch and started performing endoscopic procedures in their respective institutions in 2015–2016. The otolaryngology surgeon (M.B) has 32 years of experience in the management of ZD. An additional group of patients were assessed over the same period of the study who had undergone primary open transcervical surgical myotomy by the same surgical operator.

### Procedures

#### Flexible diverticulotomy

FD is performed with a standard adult gastroscope under a general anaesthetic with a transparent distal attachment. The muscular septum of the pharyngeal pouch is identified with or without the use of an overtube, which was only utilised in a select number of early cases. A wire may be placed into the stomach under direct vision to maintain reference to the oesophageal lumen and if necessary, place a bougie to stabilise the septum. A small mucosal incision is then made and a septotomy performed to the base of the pouch with a stag beetle (SB) knife or Zimmon needle knife. The mucosal defect is then closed with through-the-scope haemostasis clips. Patients are admitted overnight for observation and discharged the following day on a modified diet for seven days.

#### Zenker peroral endoscopic myotomy

Z-POEM is performed with a standard adult gastroscope under a general anaesthetic with a distal transparent attachment. First, the pouch and cricopharyngeal bar are isolated and a submucosal injection is placed 1–2 cm proximal to the septum. A 1.5-cm mucosotomy is performed to gain access to the submucosal space using an electrosurgical endoscopic submucosal dissection knife (IT NanoKnife or Dual-J knife). Submucosal fibres are dissected in a process known as ‘tunnelling’ on either side of the septum until it is fully exposed. Once exposed, a myotomy is performed of the septum and extended to the base of the pouch on the oesophageal side. Once completed, the remaining mucosal bridge may be dissected to prevent formation of a bar before closure with through-the-scope haemostasis clips. Peri-procedural antibiotic use is not routine. Patients are admitted overnight for observation and discharged the following day on a modified diet for seven days.

#### Rigid diverticulotomy

RD is performed using a diverticuloscope under a general anaesthetic. With the neck in hyperextension, the muscular septum of the pharyngeal pouch is identified and the diverticuloscope placed either side of the septum until the bottom of the diverticulum is exposed. A linear endoscopic stapler can then be introduced through the diverticuloscope down to the septum. A septotomy is performed with simultaneous cutting and sealing of the oesophageal and diverticular walls with at least a double row of staples. For stapling, a diverticulum of at least 2 cm is usually required as smaller pouches pose a challenge to stapling. Alternatively, the septotomy may be performed using carbon dioxide (CO2) laser-assisted stapling or the use of laser alone. During this technique, an operating microscope attached to a CO2 laser micromanipulator is used to focus the laser beam at 5–10 Watts on the muscular bridge to transect the septum. Peri-procedural antibiotic use is not universal. After RD, patients are admitted overnight for observation and discharged and on a modified diet for seven days if they remain well between post-operative days 1–3.

#### Open transcervical surgical myotomy

Open surgical repair of ZD is initially based on obtaining visualisation of the pouch through transcervical access. Patients undergo a general anaesthetic in the supine position with the neck in hyperextension and slight right tilt. A left lateral neck incision is made ventral to the sternocleidomastoid. After initial dissection, the same muscle is retracted alongside the carotid sheath, larynx, and thyroid to expose the cervical oesophagus and pouch. The pouch is dissected from surrounding connective tissue and a 5-cm myotomy performed with endostapling. After myotomy, the pouch is either excised (diverticulectomy), retracted with suturing (diverticulopexy), or inverted and oversewn. Post-procedure a drain is placed, the subcutaneous space and platysma borders sutured, and the skin incision is closed. The patient is admitted overnight and treated with antibiotics for 5–7 days. The drain is removed in 24–48 h and the patients are discharged on a modified diet.

### Primary efficacy endpoints

The primary outcomes were technical success and clinical success of each procedure. Technical success was defined as completion of all steps of the endoscopic myotomy. Clinical success was defined as reduction in Dakkak and Bennett dysphagia score (DBS) to ≤ 1 (or 0 if the pre-treatment score was 1) without need for repeat intervention. DBS was assessed at each scheduled follow-up post-procedure. The DBS score at last known follow-up was used to determine clinical success. DBS is a simple dysphagia score based on the patients’ reported symptoms that is graded from 0 to 4 (0 = no dysphagia, 1 = dysphagia to solids, 2 = dysphagia to semi-solids, 3 = dysphagia to solids and liquids, 4 = aphagia).

### Secondary efficacy endpoints

The secondary outcomes were procedure time, inpatient stay, 30-day adverse event rate, initial clinical success, and need for re-intervention.

### Statistical analysis

Data analysis was performed using Stata/MP statistical software package (StataCorp LLC, Texas, USA, Version 17). Continuous variables are presented as mean and standard deviation or median and inter-quartile range (IQR). Categorical variables are presented as counts with percentages and 95% confidence intervals (CIs). Data were analysed using chi-squared, ANOVA, or Kruskal–Wallis test depending on data type and distribution. On follow-up analysis, patients were censored at the point of failure. Univariable and multivariable logistic regression were conducted to determine variables predictive of clinical success. A *P* value < 0.05 was considered statistically significant.

## Results

### Patient cohort

In total, 126 consecutive patients underwent endoscopic treatment for ZD during the study period with no significant difference between baseline characteristics (Table 1). The median age was 74 (IQR 68–79), 31.8% (*N* = 40) were female, and the median Charlson co-morbidity index (CCI) was 3 (IQR 2–4). The mean pouch size was 37.6 mm (SD 17), median dysphagia score 2 (IQR 1–3), and 40 patients (31.8%) had previously undergone attempted management of ZD with surgery or rigid endoscopic stapling.

**Table 1:** Baseline patient characteristics

	Total	Z-POEM	FD	RD	*p*
Number (*n*)	126	45	31	50	
Age (IQR)	74 (68–79)	76 (71–80)	73 (67–81)	72 (67–79)	0.58
Female sex (%)	40 (31.8)	18 (40)	7 (22.6)	15 (30)	0.26
Charlson co-morbidity index (IQR)	3 (2–4)	3 (3–5)	3 (3–4)	3 (2–4)	0.57
Pouch size in mm (SD)	37.6 (17.0)	39.4 (15.2)	33.3 (17.6)	39.0 (18.2)	0.51
Prior aspiration pneumonia (%)	17 (13.5)	6 (13.3))	5 (16.1)	6 (12)	0.87
Previous treatment (%)	40 (31.8)	15 (33.3)	10 (32.3)	15 (30.0)	0.94
Pre-treatment DB score (IQR)	2 (1–3)	2 (1–3)	1.5 (1–3)	2 (1–2)	0.65

*DB* Dakkak and Bennett, *FD* flexible diverticulotomy, *IQR* interquartile range, *RD* Rigid diverticulotomy, *SD* Standard deviation, *Z-POEM* Zenker Peroral Endoscopic Myotomy

*p* < 0.05 is significant

*p* values determined using one-way ANOVA or Kruskal–Wallis test for continuous or ordinal variables and chi-squared for binary or categorical variables

### Procedural outcomes

**Fig. 1:**
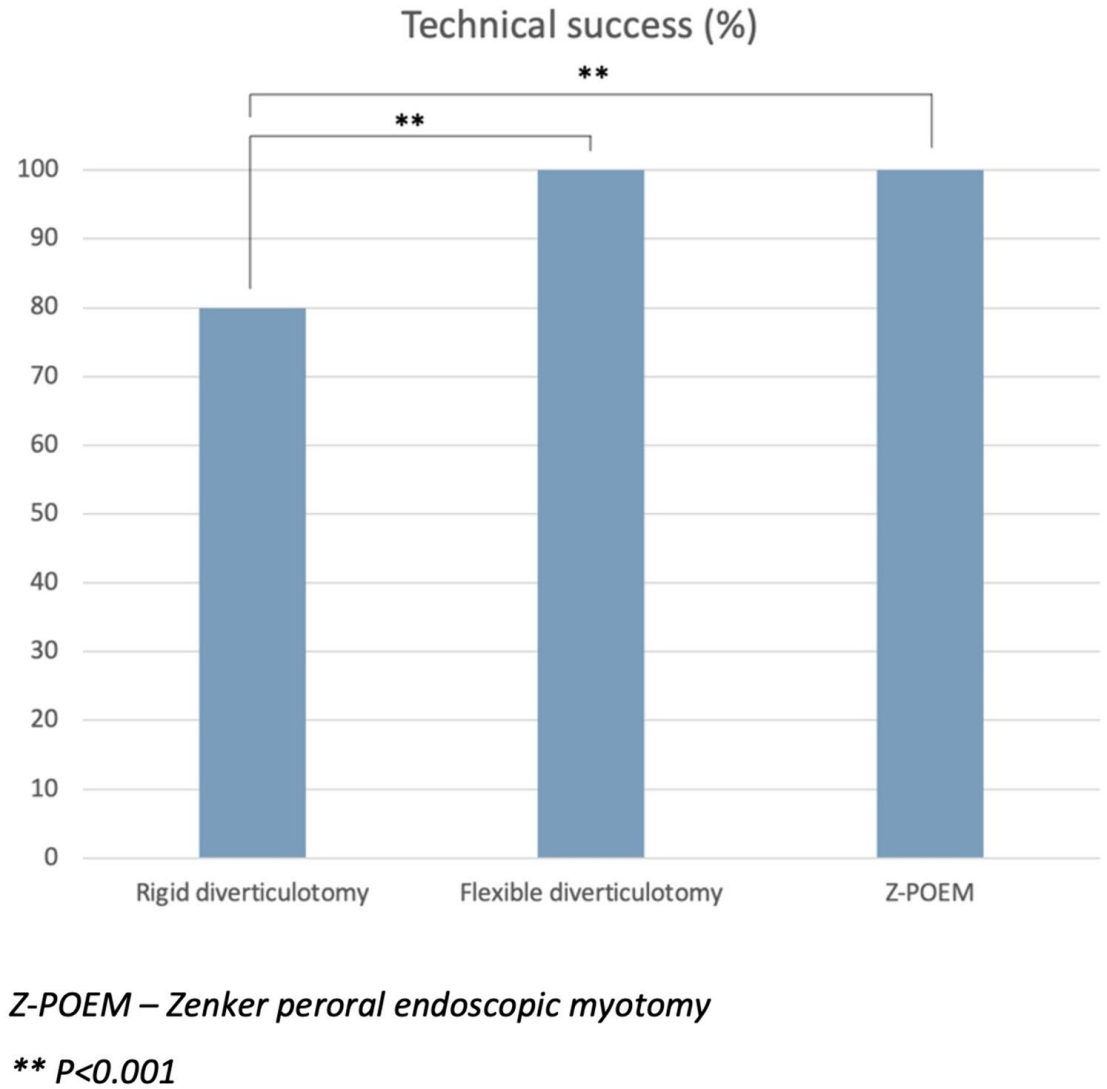
Technical success for each endoscopic technique

The procedural outcomes amongst patients who underwent Z-POEM (*n* = 45), FD (*n* = 31), or RD (*n* = 50) are summarised in Table 2. Amongst those proceeding to RD, 25 underwent endoscopic stapling only, 20 had endoscopic stapling combined with CO2 laser, and five had laser only. There was no significant difference in the operation time (*p* = 0.06), although FD was numerically quicker at an average 37.2 min with a trend towards significance. The procedure was technically successful in 100% of flexible endoscopic cases (*N* = 86) but only 80% of RD, which was significantly lower (*p* < 0.001; Fig. 1). Amongst these, the time range of the initial failed attempt was distributed widely across the cohort (2013–2021). The median inpatient stay across all three groups was 1 (*p* = 0.31), and there was no significant difference in the rate of 30-day complications (*p* = 0.98). Amongst those with failed RD, seven proceeded to open myotomy at the same procedure, two underwent balloon dilatation with and without Botox injection of the upper oesophageal sphincter, and one was referred for a flexible endoscopic approach.

**Table 2:** Procedural outcomes for the endoscopic management of Zenker’s diverticulum

	Z-POEM (*N* = 45)	FD (*N* = 31)	RD (*N* = 50)	
Operation time in mins (SD)	50.6 (17.0)	37.2 (14.6)	53.7 (9.3)	*p* = 0.06
Technical success (%)	45 (100)	31 (100)	40 (80)	*p* < *0.001*
Inpatient stay in days (IQR)	1 (1)	1 (1)	1 (1)	*p* = 0.31
30-day complications (%)	3 (6.8)	2 (6.7)	3 (7.7)	*p* = 0.98

*FD* flexible diverticulotomy, *IQR* interquartile range, *RD* Rigid diverticulotomy, *SD* Standard deviation, *Z-POEM* Zenker Peroral Endoscopic Myotomy

*p* < 0.05 is significant

*p* values determined using one-way ANOVA or Kruskal–Wallis test for continuous or ordinal variables and chi-squared for binary or categorical variables

### Clinical outcomes

**Fig. 2:**
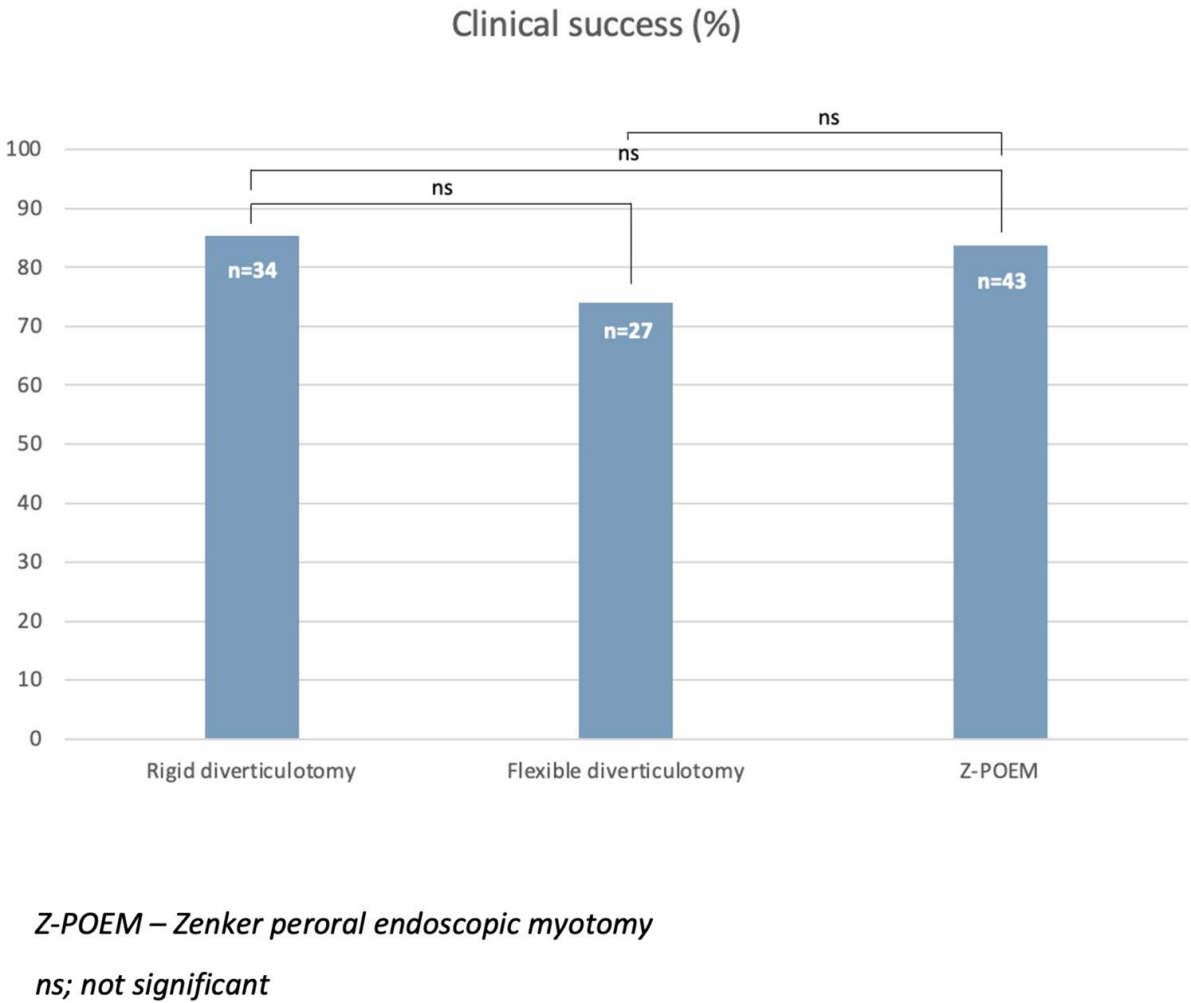
Clinical success for each endoscopic technique

The rate of clinical success across the whole cohort was 81.7% (95% CI 74.2–89.3) over a mean follow-up of 11.0 months (95% CI 8.2–13.9). Twenty-two patients were not included in the final analysis due to failed initial procedure (*n* = 10), patient death from an unrelated cause (*n* = 3), or lost to follow-up (*n* = 9). There was no significant difference in clinical success across the three endoscopic modalities (*p* = 0.48; Table 3). Clinical success was 83.7% (*n* = 43) over a median follow-up of six months (IQR 3–11) for Z-POEM, 74.1% (*n* = 27) over a median follow-up of five months (IQR 2–15) for FD, and 85.3% (*n* = 34) over a median follow-up of 10 months (IQR 1–24) for RD (Fig. 2).

**Table 3:** Clinical outcomes for the endoscopic management of Zenker’s diverticulum

	Z-POEM	FD	RD	*P*
Post-treatment DB score (IQR)	0 (0–1)	0 (0–1)	0 (0)	0.44
Early clinical success^a^ (%)	33 (94.3)	10 (76.9)	22 (95.7)	0.18
Total clinical success (%)	36 (83.7)	20 (74.1)	29 (85.3)	0.48
Recurrence (%)	3 (8.3)	4 (20)	5 (17.2)	0.50
Follow-up in months (IQR)	6 (3–11)	5 (2–15)	10 (1–24)	0.41
Repeat procedure (%)	4 (8.9)	4 (13.3)	3 (7.7)	0.72

*DB* Dakkak and Bennett, *FD* flexible diverticulotomy, *IQR* interquartile range, *RD* Rigid diverticulotomy, *Z-POEM* Zenker Peroral Endoscopic Myotomy

^a^Clinical success measured at 3 months where available

*p* < 0.05 is significant

*p* values determined using Kruskal–Wallis test for continuous or ordinal variables and chi-squared for binary or categorical variables

Early clinical success, defined as the resolution of symptoms based on DB score at three-month follow-up, was not significantly different between Z-POEM (94.3%), FD (76.9%), and RD (95.7%) amongst 65 patients with follow-up data available (*p* = 0.18). When only assessing patients without previous intervention, overall clinical success was 91.4% over a mean follow-up of 7.6 months (range 1–86) with no difference between modalities (*p* = 0.46). There was no significant difference in the rate of recurrence between the groups (RD 17.2%, FD 20.0%, Z-POEM 8.3%; *p* = 0.5). A repeat procedure was required in 11 patients (9.7%) who had initial technical success, which included RD (*n* = 3), FD (*n* = 2), Z-POEM (*n* = 4), or balloon dilatation (n = 1). The need for a repeat procedure (*p* = 0.72) or the success of the subsequent procedure was not significantly different between groups (*p* = 0.54).

We also assessed for any difference in clinical success based on the timing of the procedure. We defined an ‘early cohort’ as procedures before 2019 and a ‘late cohort’ as procedures conducted from 2019 onward. Across all patients, there was no significant difference in clinical success between early (83.3%; *n* = 42) and late cohorts (80.6%; *n* = 62), respectively (*p* = 0.728). When differentiating by procedure type, there was again no significant difference in both the early (FD 83.3%, *n* = 12 vs RD 83.3%, *n* = 30; *p* = 1.000) and late cohorts (FD 66.7%, *n* = 15 vs RD 100%, *n* = 4 vs Z-POEM 83.7%, *n* = 43; *p* = 0.212), although Z-POEM was only introduced into institutions after 2018.

### Adverse events

Amongst all three groups, the pooled rate of adverse events related to the procedure was 7.1% (95% CI 2.3–12.0). Between groups, the 30-day rate of complications was 6.8% (*n* = 3) for Z-POEM, 6.7% (*N* = 2) for FD, and 7.7% (*N* = 3) for RD, which was not significantly different (*p* = 0.98; Table 2). In the Z-POEM group, there were two post-operative chest infections and one leak. The leak was managed conservatively with nasogastric feeding and intravenous antibiotics over a 28-day admission with complete recovery. In the FD group, there was one chest infection and one perforation associated with collection. This patient was readmitted 48 h after the original procedure and had evidence of mediastinitis. They required wash-out in theatre, external drainage, and nasojejunal feeding over a 30-day admission. There was evidence of complete recovery and improvement in DB score to 1 (baseline 2) over 15-month follow-up. In the RD group, there were three oesophageal perforations managed conservatively with an average length of stay of 9.7 days. In addition, there were three cases of dental damage (7.3%) compared to zero cases with flexible endoscopic methods that trended towards significance (*p* = 0.06). Across the whole cohort the overall mortality was 11.1% (*n* = 14), but none were attributed to the procedure or aspiration pneumonia.

### Variables predictive of success

We conducted a logistic regression analysis to determine whether any variable was predictive of clinical success after endoscopic treatment. On univariable logistic regression, no variables were predictive of early or overall clinical success. Interestingly, previous pouch treatment trended towards significance (OR 3.2; 95% CI 0.87–11.9; *p* = 0.08), whereas pouch size had no impact (OR 0.99; 95% CI 0.96–1.01; *p* = 0.50). On multivariable logistic regression, this potential correlation with previous treatment was not seen after backward stepwise regression.

### Surgical outcomes

Over the same period, 11 patients underwent open transcervical surgical myotomy that included four patients undergoing primary surgery and seven after failed RD. The outcomes for these patients are summarised in Table 4. The median age was 69 (IQR 68.5–73), 45.5% (*n* = 5) were female, and the median CCI was 3 (2.5–4). The average pouch size was 53.5 mm (SD 10.8; *n* = 4; 7 missing), seven (63.6%) had undergone previous endoscopic or surgical treatment, and the median pre-treatment DB dysphagia score was 2. Overall, the clinical success was 100%, median inpatient stay was 2 days (IQR 1–2), and early clinical success at 3 months was 90% (*n* = 9). The mean operation time was 180 min (SD 10; *n* = 3, 8 missing), and 30-day complication rate was 27.3%, which included an oesophageal perforation with primary repair, evacuation of a haematoma, and transient ischaemic attack. Finally, over a median follow-up of nine months (IQR 3–13), the clinical success was 36.4% (*n* = 4) with five patients (45.5%) requiring a repeat intervention.

**Table 4:** Baseline characteristics, procedural, and surgical outcomes amongst those undergoing open surgical myotomy

	Surgical myotomy
Number (*n*)	11
Age (IQR)	69 (68.5–73)
Female sex (%)	5 (45.5)
Charlson co-morbidity index (IQR)	3 (2.5–4)
Pouch size in mm (SD)	53.5 (10.8)
Previous treatment (%)	7 (63.6)
Pre-treatment DB score (IQR)	2 (2)
Operation time in mins (SD)	180 (10)
Technical success (%)	11 (100)
Inpatient stay in days (IQR)	2 (1–2)
30-day complications (%)	3 (27.3)
Early clinical success^a^ (%)	9 (90)
Clinical success (%)	4 (36.4)
Follow-up in months (IQR)	9 (3–13)
Repeat procedure (%)	5 (45.5)

*DB* Dakkak and Bennett, *IQR* Interquartile range, *SD* Standard deviation

^a^Clinical success measured at three months where available

## Discussion

This study compared the safety and efficacy of three endoscopic techniques for the management of ZD. We have shown that amongst 126 patients undergoing endoscopic treatment, the clinical efficacy of the procedure was 81.7% over a mean follow-up of 11.0 months with no significant difference between the chosen technique (RD 85.3%, FD 74.1%, Z-POEM 83.7%; *p* = 0.48). The similarity between groups held true for both early clinical success and need for repeat procedure. The main difference between techniques was the significantly lower technical success of RD (80%, *n* = 40) when compared to flexible endoscopic approaches that were both successful in 100% of cases (*p* < 0.001). The lower rate of technical success can be explained by the nature of RD requiring good neck extension and endoscopic access through a diverticuloscope. The difficulty faced is exemplified by the rate of dental damage in this cohort compared to flexible methods (7.3% vs 0%; *p* = 0.06). The concern is that a failed RD is often converted to an open surgical myotomy under the same anaesthetic. However, we have shown that open surgical myotomy is associated with a longer median inpatient stay (2 days; *p* = 0.05), high rate of adverse events (27.3%; *n* = 3) and significantly lower clinical success (36.4%; *p* < 0.01) over comparable median follow-up (9 months; IQR 3–13; *p* = 0.53). Therefore, conversion to an open approach may not be considered appropriate without an attempt at a flexible endoscopic technique.

In the literature, there have been limited publications on the comparison of rigid and flexible endoscopic techniques. Al Ghamdi et al. [11] looked at 245 patients undergoing endoscopic management of ZD and found no significant difference in the technical success (RD 87.5%; FD 95.3%; Z-POEM 95%; *p* = 0.18) or clinical success (RD 89.2%; FD 86.7%; Z-POEM 92.7%; *p* = 0.26) between procedures over a mean follow-up of 282 days (SD 300.5), although technical success was proportionally lower with RD. The rate of adverse events was significantly lower with FD (2.3% vs 30.0% RD and 16.8% Z-POEM; *p* < 0.05), whereas we found that adverse events were not significantly different between groups (*p* = 0.98) with a pooled rate of 7.1%. In a single-centre study by Wallerius et al. [12], they compared 424 patients who underwent RD (*n* = 267), FD (*n* = 70), or open myotomy (*n* = 87). FD was associated with a higher rate of recurrence (17.1%) compared to either RD with laser (4.2%) or open myotomy (1.1%) but not RD with stapling (17.4%). In addition, FD was associated with a significantly higher rate of procedural complications (18.6%; *p* < 0.01) compared to other modalities. However, in this study the development of subcutaneous emphysema was one of the criteria used to define perforation. Whilst this is not uncommon with flexible endoscopic techniques, it does not necessarily correlate with a clinically significant adverse outcome as evidenced by the two patients requiring surgical intervention for perforation in their study undergoing RD. Furthermore, meta-analysis data from eight retrospective studies (including the study by Wallerius et al. [12]) involving 1281 patients, showed comparable rates of clinical success (risk difference 0.07; 95% CI − 0.05 to 0.19; *p* = 0.26), technical success (risk difference 0.07; 95% CI − 0.03 to 0.16; *p* = 0.18), and serious adverse events (risk difference − 0.03; 95% CI − 0.13 to 0.07; *p* = 0.052) between flexible and non-flexible techniques [13]. This held true on subgroup analysis comparing flexible techniques with RD or surgery alone.

Collectively, it appears that with good access to the pouch, all three endoscopic procedures can provide a meaningful septotomy with improvement in swallowing on follow-up. Furthermore, we can see that amongst those undergoing FD, there was a lower proportion of early clinical success (76.9%), overall clinical success (74.1%), and need for repeat procedure (13.3%). Although this was not statistically significantly, it provides further evidence that the important aspect of the procedure is ensuring a complete myotomy that may be easier to achieve with RD or Z-POEM. The proportionally lower efficacy of FD could be due to an inadequate number of cases. This is supported by the more comparable results in the larger retrospective series by Al Ghamadi et al. [11]. However, the size of the pouch amongst patients undergoing FD in their cohort was significantly smaller at 28.6 mm, and more patients were treatment naïve that would reduce procedural difficulty. Another possibility is the predominant move to Z-POEM from 2019 onwards in our cohort. Endoscopists performing Z-POEM will have gained invaluable experience on the management of ZD when performing FD, and the learning curve for Z-POEM is likely to be lower given the transferrable skills with traditional POEM for achalasia. Taken collectively, this shows that patient factors (e.g. prior intervention, pouch size), and endoscopic factors (e.g. procedural experience) may influence clinical efficacy beyond simply the procedure choice.

Previous studies have looked at the comparison between FD and Z-POEM. In one prospective study by Swei et al. [14], 28 patients underwent FD or Z-POEM with comparable technical success (FD 100% vs Z-POEM 100%), clinical success (FD 86.7% vs Z-POEM 100%; *p* = 0.18), and adverse events (FD 6.7% vs Z-POEM 0%) during follow-up, which was consistent at 1 year. In this study, the procedure time was numerically longer during FD (60.2 ± 22.4) than Z-POEM (43.9 ± 13.7; *p* = 0.19). This differs from two published abstracts comparing a small cohort of patients with ZD who underwent FD or Z-POEM. In each study, Z-POEM was associated with a longer procedure time, lower rate of adverse events, and higher clinical success [15, 16]. Amongst all these studies, what is not accounted for is the skill set of the endoscopist (e.g. trained in third space) and the degree of endoscopic difficulty due to access, previous treatment, or submucosal fibrosis. In these situations, the proposed advantage of Z-POEM is that it attains better endoscopic access, isolation of the muscular septum, and more complete myotomy.

The retrospective nature of our study is associated with some limitations. We used a relatively simple dysphagia score (DBS) to determine clinical success as it enabled a more consistent analysis of retrospective data. This may not account for other features of ZD, including regurgitation. The follow-up duration, whilst consistent between groups, was relatively short at 11 months. A few patients were lost to follow-up, particularly amongst patients undergoing RD, which may limit the interpretation of results. All RD procedures were performed by a single operator and grouped collectively regardless of the technique due to patient numbers.

In summary, this was a multicentre study comparing three endoscopic modalities for the treatment of ZD. We have shown that RD, FD, and Z-POEM are all safe and effective treatment options for the resolution of symptoms with comparable rates of adverse events. However, rigid approaches are associated with a significantly lower technical success that is often converted to open surgical myotomy. Given the increased healthcare utilisation and higher complication rates of open neck surgery, a failed RD approach should prompt a referral for a flexible approach by an experienced practitioner. Flexible techniques could be considered first-line treatment for ZD as they are associated with high technical and clinical success. However, they should be conducted by endoscopists with experience in third space endoscopy to optimise septotomy, reduce the risk of adverse events, and ultimately enhance long-term clinical success. Given the rarity and growing complexity of ZD treatment, decision-making on treatment options may benefit from multidisciplinary input.
